# Emerging and Re-Emerging Pathogens in Valvular Infective Endocarditis: A Review

**DOI:** 10.3390/pathogens13070543

**Published:** 2024-06-27

**Authors:** Maximilian Reisinger, Mateusz Kachel, Isaac George

**Affiliations:** 1 Division of Cardiac, Thoracic & Vascular Surgery, New York-Presbyterian Hospital, Columbia University Medical Center, New York, NY 10032, USA; 2 Center for Cardiovascular Research and Development, American Heart of Poland, 40-028 Katowice, Poland

**Keywords:** infective endocarditis (IE), native valve endocarditis (NVE), prosthetic valve endocarditis (PVE), pathogens

## Abstract

Infective endocarditis (IE) is a microbial infection of the endocardial surface, most commonly affecting native and prosthetic valves of the heart. The epidemiology and etiology of the disease have evolved significantly over the last decades. With a growing elderly population, the incidence of degenerative valvopathies and the use of prosthetic heart valves have increased, becoming the most important predisposing risk factors. This change in the epidemiology has caused a shift in the underlying microbiology of the disease, with Staphylococci overtaking Streptococci as the main causative pathogens. Other rarer microbes, including *Streptococcus agalactiae*, *Pseudomonas aeruginosa*, *Coxiella burnetti* and *Brucella*, have also emerged or re-emerged. Valvular IE caused by these pathogens, especially *Staphylococcus aureus*, is often associated with a severe clinical course, leading to high rates of morbidity and mortality. Therefore, prompt diagnosis and management are crucial. Due to the high virulence of these pathogens and an increased incidence of antimicrobial resistances, surgical valve repair or replacement is often necessary. As the epidemiology and etiology of valvular IE continue to evolve, the diagnostic methods and therapies need to be progressively advanced to ensure satisfactory clinical outcomes.

## 1. Introduction

Infective endocarditis (IE) is a rare but potentially fatal disease that is associated with significant morbidity and mortality [1]. The pathogenesis involves an inflammatory reaction of the endocardial surface caused by infection through varying pathogens [2]. In the majority of cases, the heart valves, both native and prosthetic, are involved [3]. Timely diagnosis and treatment of valvular IE are crucial to prevent the feared complications, specifically valve dysfunction leading to heart failure (HF) and thromboembolic stroke [4,5]. Additionally, with an in-hospital and 1-year mortality rate of 20% and 37%, respectively, the importance of prompt recognition and adequate management is further emphasized [6]. 

A changing pattern of risk factors, in particular a decrease in rheumatic heart disease and an increase in degenerative valvopathies and prosthetic valves, as well as an increasing incidence of antibiotic resistances, has caused the microbiology of valvular IE to evolve [7,8]. Over the last decades, there has been a shift from predominantly streptococci to now staphylococci being the most common causative agents [9]. Also, new pathogens that lead to valvular IE have emerged and make both the diagnosis and treatment increasingly challenging [9]. In addition to the current guidelines, the peculiarities of re-emerging as well as new emerging pathogens need to be considered to guide ideal management [10,11].

The aim of this review is to present the most pertinent underlying pathogens of native valve endocarditis (NVE) and prosthetic valve endocarditis (PVE). Moreover, specific diagnostic methods and treatment in the context of these pathogens are discussed. As IE is such a rapidly evolving disease, this review offers a current update of its status to increase awareness of emerging and re-emerging pathogens and guide clinicians in timely recognition and ideal management. 

## 2. Epidemiology

**Figure 1:**
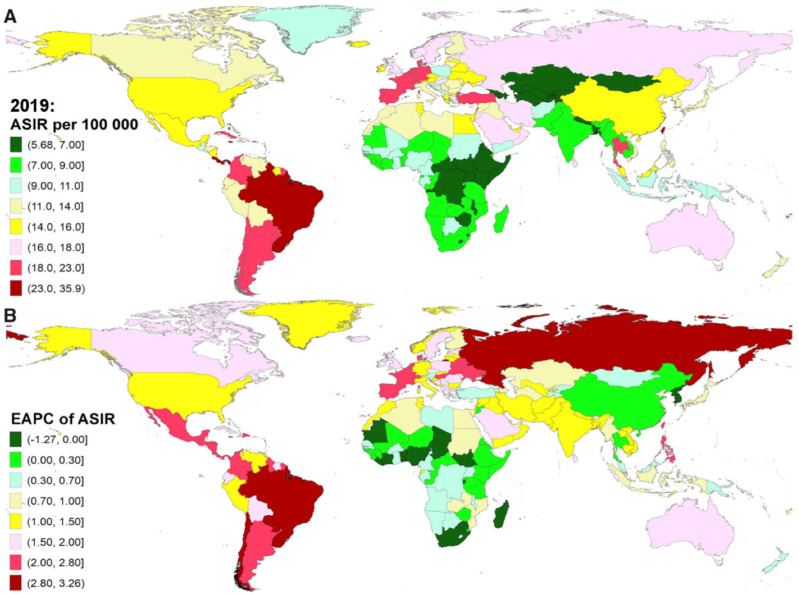
Global incidence of infective endocarditis (IE). (**A**) Age-standardized incidence rate (ASIR) of IE in 2019. (**B**) Estimated annual percentage change (EAPC) in ASIR of IE from 1990 to 2019. Reprinted with permission from Yang et al. [13].

Over the last 30 years, the epidemiology of IE has changed significantly. The analyses of the Global Burden of Disease Study aimed to estimate the temporal trends globally and in different regions from 1990 to 2019 [12,13,14]. Overall, the incidence and mortality of IE have continued to rise in the past 30 years, especially in higher socio-demographic-index (SDI) regions (Figure 1). Also, a shift in the population affected (moving from the young to the elderly) was seen. Globally, patients aged 50 years or older accounted for nearly 63% of IE incidence and 79% of IE mortality in 2019, significantly outnumbering the incidence and mortality in 1990 (35% and 60%, respectively).

In 1990, there were 478,002 cases of IE reported globally, which increased to 1,090,527 in 2019. Similarly, the age-standardized incidence rate (ASIR) rose from 9.91 to 13.8 per 100,000 person years over the past 30 years. Consequently, an increase in mortality was reported with over 66,322 deaths reported in 2019 (28,754 in 1990) and in age-standardized mortality rate (ASMR) to 0.87 (0.73 per 100,000 person years in 1990). When considering the disability-adjusted life years (DALYs), a 1.5 times increase was noted (1.12 to 1.72 million). However, the age-standardized DALYs rate (ASDR) decreased from 22.78 in 1990 to 21.93 per 100,000 person years in 2019 [12,13].

The analysis performed by Yang et al. showed that the highest incidence rate of IE was seen in the high-middle/high SDI regions across all years from 1990 to 2019: 11.34/11.01 per 100,000 person years in 1990 and 15.86/15.85 per 100,000 person years in 2019 [13]. Conversely, the low SDI regions experienced an event rate of 7.39 per 100,000 person years in 2019. The Global Burden of Disease Study 2019 showed that Tropical Latin America, Southern Latin America and Caribbean were among the top three regions for the highest ASIR in 2019 (18.72 to 24.25 per 100,000 person years). On the contrary, the top three GBD regions with the lowest ASIR in 2019 were Central Asia, Eastern Sub-Saharan Africa and Central Sub-Saharan Africa (6.35 to 6.89 per 100,000 person years). Nevertheless, a significant increase in the number of cases was reported across all SDI regions. These differences may be partially explained by the higher percentage of elderly people in high SDI regions, who tend to present with comorbidities, such as cancer or diabetes, or undergo hemodialysis, which predisposes to IE incidence. Also, the frequent use of prosthetic valves and implantable devices plays a significant role [15,16,17,18]. Contrarily, in low SDI regions, a rapid rise in IE was halted with widespread introduction of antibiotics that limited the rheumatic heart disease incidence. However, improvement in diagnostic methods and accessibility to healthcare did not follow, thus contributing to the substantial underreporting of cases.

Similarly, the highest mortality in 2019 was observed in high SDI regions (1.16 per 100,000 person years), followed by low SDI (0.79 per 100,000 person years) and middle SDI regions (0.62 per 100,000 person years). The largest increase in ASMR was observed in a high SDI region (estimated annual percentage change (EAPC) = 2.01), followed by a high-middle region (EAPC = 0.62), whereas middle SDI and low SDI regions presented a downward trend in the ASMRs (lowest EAPC = −0.62 in the middle SDI region). High mortality in developed regions reflects both the nominal increase in the number of cases, as well as a population structure with great portion of old and more fragile people who are more susceptible to IE. Moreover, the dominant role of *Staphylococcus aureus* in IE etiology in these regions translates into more antibiotic-resistant strains, thus limiting the treatment options. In low SDI regions, a disproportionately high (to the number of cases) mortality is caused by the low diagnostic capabilities and inaccessible healthcare.

## 3. Pathogenic Spectrum in Valvular Infective Endocarditis

**Figure 2:**
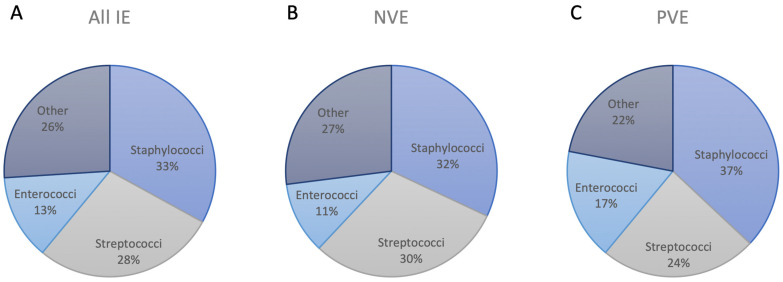
Pathogenic spectrum of infective endocarditis (IE). Relative distribution of underlying pathogens in (**A**) all IE, (**B**) native valvular endocarditis (NVE) and (**C**) prosthetic valvular endocarditis (PVE) [1,3,20,22,28].

Along with the shift in the epidemiology of IE, the underlying microbiology of the disease has changed too [19]. For all valvular IE, staphylococci, streptococci and enterococci comprise the majority of causal microbes with ~80% [1,20]. While there has been a steady increase in IE cases due to both *Staphylococcus* and *Streptococcus* spp., staphylococci, especially *Staphylococcus aureus*, have overtaken *Streptococcus viridans* as the leading causative agents in the 21st century [21,22]. The incidence of *Enterococcus* spp. cultured in valvular IE has also shown a steady rise, especially in the setting of elderly patients and transcatheter aortic valve replacement (TAVR) [23,24]. Other important pathogens include the HACEK (*Haemophilus* spp., *Aggregatibacter actinomycetemcomitans*, *Cardiobacterium hominis*, *Eikenella corrodens* and *Kingella kingae*) organisms, aerobic Gram-negative bacilli and fungi, specifically *Candida* and *Aspergillus* spp., accounting for ~5–10% of all valvular IE cases [20,25,26]. The HACEK organisms are a group of Gram-negative, fastidious bacteria that predominantly affect younger patients and show a slight predilection for prosthetic heart valves, even though they are associated with both NVE and PVE [25,27]. Fungal endocarditis predominantly affects patients in an immunocompromised state and is most frequently associated with PVE [26]. The distribution of pathogens isolated in cases of all valvular IE, NVE and PVE is shown in Figure 2.

In the setting of NVE, the majority of cases are also due to staphylococci, streptococci and enterococci, accounting for 30–40%, 25–35% and 10–15%, respectively [3,20,28]. From the *Staphylococcus* spp., *Staphylococcus aureus* presents the main causative agent with ~30%, while coagulase-negative Staphylococci are cultured in ~5–10% of NVE cases [3,28]. It is important to note that the relative contribution of *Staphylococcus aureus* increases to ~60–90% when considering right-sided NVE only [29]. This can be largely explained by the increased association of intravenous drug use, central venous catheters and intracardiac devices with right- compared to left-sided NVE [29,30]. From the *Streptococcus* spp., *Streptococcus viridans* is the major infectious agent with ~15–20%, while other streptococci, specifically *Streptococcus gallolyticus*, account for ~10–15% [3,20,28]. Whereas *Staphylococcus aureus* has replaced *Streptococcus viridans* as the main underlying pathogen in all valvular IE, some studies show streptococci as the leading cultured microbe in left-sided NVE [31,32]. Moreover, an increased number of streptococci and a decreased number of staphylococci have been associated with bivalvular compared to monovalvular left-sided NVE [32]. Enterococci represent the third most frequent group of organisms causing NVE, mainly affecting the left-sided heart valves [3,20,28]. 

In the setting of PVE, staphylococci remain the main causal microbes with 30–40% [3,22,28]. However, the relative contribution of *Staphylococcus aureus* decreases and that of coagulase-negative staphylococci increases to ~20–25% and ~15–20%, respectively [3,22,33]. This demonstrates the predilection of coagulase-negative staphylococci to prosthetic heart valves [33]. Streptococci and enterococci are the second and third most common underlying pathogens, with each accounting for ~15–25% [3,22,28]. Important differences exist when considering PVE following surgical aortic valve replacement (SAVR) and TAVR separately from another. Many studies have reported enterococci as the leading cause of TAVR-PVE, with an incidence of up to 25% [34,35,36]. The potential reason that has been suggested for these findings is that most TAVR cases are performed through a transfemoral approach, with this pathogen having a predilection for the groin area [37]. The majority of SAVR-PVE is caused by staphylococci, with an incidence of up ~60%, as reported in an analysis of the Placement of Aortic Transcatheter Valves (PARTNER) trials [38]. This was the first study to directly compare the microbiological profile of TAVR- and SAVR-PVE, and it was also the first report that did not show the increased incidence of enterococci in PVE following TAVR, with the majority of cases due to staphylococci and streptococci [38]. 

In addition to the major pathogens, there are also rare and emerging microbes that warrant consideration when facing valvular IE. Mainly affecting the native left-sided heart valves, *Streptococcus agalactiae* is a rare causative bacterium [39]. With an approximate incidence of 2–3% in left-sided NVE, the severity of valvular IE due to this pathogen has been shown to be similar to *Staphylococcus aureus* [39]. Another rare but important microbe, originally mainly associated with right-sided but now also increasingly found in left-sided valvular IE, is *Pseudomonas aeruginosa* [29,40,41]. Its incidence has been increasing in right-sided valvular IE, especially in the setting of intravenous drug use [29]. However, there are also reports of left-sided valvular IE cases, which are mainly attributed to previous cardiac intervention or surgery [40,41]. Two of the most common fastidious pathogens found in culture-negative IE are *Coxiella burnetii* and, less frequently, *Bartonella* [42]. Both of these microbes mainly affect the left-sided heart valves, with a strong predilection for the aortic valve [42]. *Coxiella burnetii* has been shown to account for ~1% of valvular IE in an international cohort, and it has been predominantly associated with prior cardiac procedures involving valvular or aortic prosthesis, as well as with native valvopathies [3,43]. When considering PVE and aortic vascular graft endocarditis alone, the relative contribution of *Coxiella burnetii* can increase up to 25% [43]. Lastly, *Mycobacterium chimaera*, a non-tuberculous slow-growing mycobacterium, is a rare and fastidious emerging pathogen [44]. It is difficult to culture, making its diagnosis challenging. Similar to *Coxiella burnetii* and *Bartonella*, it mainly affects the left-sided heart valves, with the majority of cases involving the aortic valve [44]. It has been mainly found in patients who have undergone open-heart surgery, including aortic and mitral valve, as well as coronary artery bypass graft surgery [44]. 

## 4. Diagnostic Methods

The diagnosis of infective endocarditis is based on clinical manifestation supported by microbiological assessment and cardiac imaging targeting potential structural abnormalities (i.e., vegetations) [11]. The Duke Criteria are an established tool for IE diagnosis. Introduced in 1994, they underwent several modifications to incorporate novel imaging techniques and account for the changing landscape of IE and demonstrated a high overall sensitivity of 80% [45,46]. However, the initial version relied heavily on echocardiography imaging, which did not account for the highly variable spectrum of IE clinical manifestation. The limitations are particularly pronounced in the presence of prosthetic material, such as PVE, aortic grafts or cardiac devices, where the echocardiography can be normal or inconclusive in up to 30% of cases despite the presence of IE [47]. Nevertheless, both the 2015 and 2023 European Society of Cardiology (ESC) guidelines presented a revised criteria to diagnose IE that introduced multimodality imaging (echocardiography, cardiac/whole-body computed tomography (CT), cerebral magnetic resonance imaging (MRI), 18F-fluorodeoxyglucose positron emission tomography/computed tomography (18F-FDG PET-CT) and single photon emission tomography/computed tomography with technetium99m-hexamethylpropyleneamineoxime (99mTc-HMPAO-SPECT/CT)) to improve the diagnostic yield. This approach has shown to be superior over the traditional diagnostic criteria [11,47,48]. Also, recently, a new version of the Duke Criteria has been proposed by the International Society for Cardiovascular Infectious Diseases (ISCVID) that implemented significant changes, including new microbiology diagnostics (enzyme immunoassay for Bartonella species, polymerase chain reaction (PCR), amplicon/metagenomic sequencing, in situ hybridization), imaging (18F-FDG PET-CT, cardiac CT) and inclusion of intraoperative inspection as a new Major Clinical Criterion [49]. Still, the definitive method of investigation remains the examination of resected tissue or embolic fragments. Additionally, histopathological analysis may facilitate the diagnosis of non-infectious causes of endocarditis, such as neoplastic or autoimmune [50].

### 4.1. Clinical Features

Recognizing IE based on clinical manifestation can pose a serious challenge. The disease can present as an acute, rapidly progressive infection, but also as a subacute or chronic disease with low-grade or even no fever and non-specific symptoms that may mislead or confuse the initial assessment and mimic many other conditions (such as rheumatologic, neurologic and autoimmune diseases, and even malignancies). According to the EURO-ENDO registry, the most common symptoms of IE include fever (77.7%), cardiac murmur (64.5%) and congestive HF (27.2%) [3]. An atypical presentation is common in elderly or immunocompromised patients [51,52,53]. In these cohorts, the inherent presence of multiple risk factors should prompt a careful survey despite the lack of obvious symptoms. An early involvement of the endocarditis team to guide the management and evaluation of potential cardiac and non-cardiac risk factors supporting IE diagnosis is crucial.

### 4.2. Microbiological Diagnosis

Blood cultures are a mainstay of IE diagnosis, allowing for both identification and susceptibility testing. At least three sets of blood cultures are obtained at 30 min intervals prior to antibiotic therapy, each containing 10 mL of blood, and are incubated in both aerobic and anaerobic conditions [46,54]. The standard pathogen identification process may be time consuming, thus causing delays in initiating target antibiotic therapy. The matrix-assisted laser desorption/ionization mass spectrometry (MALDI-TOF MS) method has been proposed as an alternative, allowing for rapid identification in a matter of hours [55]. However, despite the technological advancements, the appropriate antibiotic therapy and susceptibility determination must be carried out according to a validated, standardized methodology [56]. Thus, the minimum inhibitory concentration (MIC) remains the method of choice. 

According to the literature, in approximately 10–20% of all endocarditis cases, a pathogen remains unidentified by blood culture [22,57]. In this scenario, when other clinical criteria for IE are met, a diagnosis of blood-culture-negative endocarditis (BCNIE) is established. BCNIE most commonly arises as a consequence of sterilized blood cultures due to previous antibiotic administration (35–74% of cases) [57]. BCNIE can also be caused by fungi or fastidious bacteria, notably obligatory intracellular bacteria. Isolation of these micro-organisms requires culturing on specialized media, serological testing or specific PCR assays [58,59].

### 4.3. Imaging

Echocardiography imaging, both transthoracic (TTE) and transesophageal (TEE), remains the first-in-line modality used to diagnose infective endocarditis [60]. Echocardiography focuses on visualizing potential vegetations, perivalvular complications (abscess, pseudoaneurysm, new partial dehiscence of prosthetic valve, leaflet perforation) and the presence of intracardiac fistulas. Importantly, vegetation size (maximal length) is a key metric that guides surgical intervention [61]. 

TEE is helpful in a wide range of clinical scenarios due to limitations of TTE to diagnose perivalvular complications, small vegetations, prosthetic valve endocarditis and vegetations associated with cardiac implantable electronic devices (CIEDs). TEE is strongly recommended in patients with an inconclusive TTE, in patients with a negative TTE and a high suspicion of IE, as well as in patients with a positive TTE, in order to document local complications. Repeating TTE and/or TEE should be considered during follow-up of uncomplicated IE in order to detect new silent complications and monitor vegetation size [11].

However, echocardiography might be unable to diagnose abnormalities in the early stages of disease or when the imaging is technically difficult, such as in patients with more than one artificial heart valve. Of note, a negative result does not exclude IE, especially in people with a moderate or high risk of endocarditis, suggesting the use of different imaging modalities. For instance, combining nuclear medicine techniques with standard visualization modalities increases diagnostic sensitivity from 52–70% to 91–97% without compromising specificity and allows for the identification of primary infection source and/or septic embolism [62].

CT imaging may help in diagnosing perivalvular and periprosthetic complications of IE (abscesses, pseudoaneurysms and fistulae) and is recommended in both NVE and PVE if TEE is not conclusive or not feasible [63,64,65]. Also, CT can help with future navigation and surgical procedure pre-planning. Moreover, a whole-body scan enables the detection of distant lesions and sources of bacteremia [66].

The use of MRI in IE is limited mainly due to its low spatial resolution and the signal interference when the prosthetic valve or implant is present [67,68]. The main indication is the diagnosis of IE-related complication of neurological system where MRI expresses higher sensitivity than CT [69].

As mentioned before, nuclear imaging greatly improves the diagnostic capabilities of IE detection. Since 2015, the diagnostic criteria for suspected PVE in patients with inconclusive echocardiography assessment include 18F-FDG PET-CT, along with 99mTc-HMPAO-SPECT/CT [70,71]. Currently, the 2023 ESC guidelines on IE recommend the use of nuclear imaging for diagnosis of IE and cardiac complications in suspected PVE in cases of inconclusive echocardiography [11].

## 5. Management and Outcomes

The mainstay of valvular IE management consists of antibiotic treatment and surgical valvular repair or replacement, in more severe cases [10,11]. Even though international guidelines are in place to guide ideal management, they are mainly based on observational cohort studies instead of randomized clinical trials (RCTs) [10,11]. In order to improve the outcomes, significantly more RCTs are needed. Furthermore, patients diagnosed with valvular IE are increasingly cared for by a multidisciplinary endocarditis team, including cardiologists, cardiothoracic surgeons and infectious disease specialists, as described in recent guidelines [72].

### 5.1. Antibiotics

All patients diagnosed with IE require antibiotic treatment following the acquisition of blood cultures [10,11]. While the results of the blood cultures are pending, empirical therapy is initiated. The choice of empirical antimicrobial drugs is primarily based on the place of infection and whether NVE or PVE is present. In community-acquired NVE or PVE occurring later than 12 months following the index procedure, a multidrug regimen consisting of ampicillin with ceftriaxone or with (flu)cloxacillin and gentamicin is recommended [11]. In patients in these settings who are allergic to beta-lactams, a combination of either cefazolin or vancomycin with gentamicin should be considered [11]. In nosocomial and non-nosocomial healthcare associated IE as well as PVE occurring within 12 months following the index procedure, a multidrug regimen including either vancomycin or daptomycin with gentamicin and rifampin is recommended [11]. Table 1 shows a summary of the empirical antimicrobial therapy, including the drug dosages and routes of administration.

According to the culture results, microbial resistance patterns, affected valve type (native or prosthetic) and the severity of the clinical course, the antibiotic treatment regimen is modified. The recommended duration of treatment in NVE and PVE is 2–6 weeks and at least 6 weeks, respectively [11]. While intravenous therapy is essential in the first 10 days, switching to oral antibiotics has been shown to be non-inferior in left-sided valvular IE in the Partial Oral Treatment of Endocarditis (POET) RCT [73]. However, it is important to note that only patients with blood cultures positive for *Streptococcus*, *Staphylococcus aureus*, coagulase-negative staphylococci and *Enterococcus faecalis* were included [73]. Therefore, if blood cultures are positive for other pathogens, especially ones leading to a more severe clinical course, continued intravenous therapy is recommended [11]. 

### 5.2. Surgery

**Figure 3:**
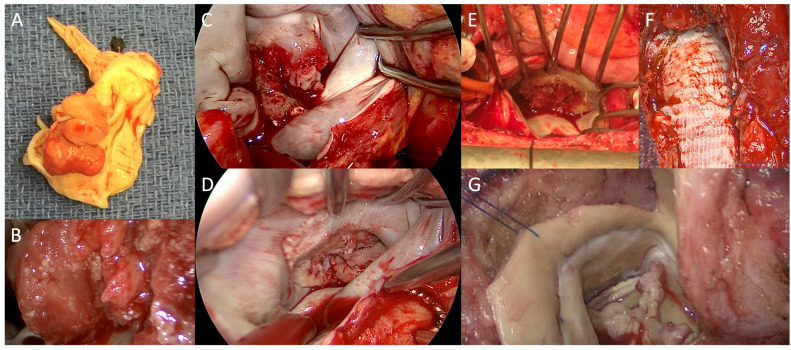
Representative intraoperative images of infective endocarditis (IE) requiring surgery. Large vegetations on the mitral valve leaflets (**A**) after excision and (**B**) in situ. (**C**–**E**) Three cases of IE affecting the native mitral valve. (**F**) A bioprosthetic aortic graft requiring explantation due to IE. (**G**) IE of a bioprosthetic aortic valve leading to severe aortic stenosis.

Out of all patients diagnosed with IE, 40–50% undergo surgical treatment [74]. The three main indications for valvular IE are valve dysfunction leading to HF, uncontrolled infection and high risk of embolization or an established embolus [10,11]. HF represents the most common surgical indication with the presence of pulmonary edema and cardiogenic shock requiring emergency surgery (<24 h). In patients with IE who develop HF, valvular surgery has been associated with a significantly decreased in-hospital and 1-year mortality rate compared to medical treatment alone (20.6% vs. 44.8% and 29.1% vs. 58.4%, respectively) [4]. With uncontrolled infection, local complications, including perivalvular abscess, fistula and pseudoaneurysm as well as life-threatening sepsis, can ensue, making this an indication for urgent surgery (3–5 days). Lastly, the presence of a vegetation with a size ≥10 mm with emboli or with another reason for surgery also represents an indication for urgent surgery (3–5 days). All the main indications for surgery in patients diagnosed with IE are summarized in Table 2. Moreover, representative intraoperative images of NVE and PVE that require surgery are shown in Figure 3. While early valvular surgery has been associated with decreased mortality, severe HF, paravalvular complications and stroke have all been associated with increased mortality [4,75,76]. 

Compared to other causative microbes, valvular IE due to *Staphylococcus aureus* has been shown to have the highest incidence of HF (42.8–62%), perivalvular abscess (30.9–60%), sepsis (23.5–51.4%), stroke (27.1–54%), systemic embolic events (33.3–63%) and acute renal failure (23.3–51.4%) [77,78,79,80]. While the incidences of HF and stroke are comparable between NVE and PVE, the rate of systemic embolic events is higher in NVE, and the rates of perivalvular abscess, sepsis and acute renal failure are higher in PVE [77,78,79,80]. Moreover, valvular IE caused by *Staphylococcus aureus* has been associated with a significantly increased in-hospital and long-term mortality rate of 25.3% and 50.4% in NVE, respectively, and 48.5% and 68.2% in PVE, respectively [77,78]. As *Staphylococcus aureus* has emerged as the main underlying pathogen in both NVE and PVE, considering timely surgical management has become increasingly important. Studies investigating the benefit of early valve surgery in patients with valvular IE caused by *Staphylococcus aureus* compared to other causative microbes have shown similar postoperative morbidity and mortality rates [79,80,81]. However, the preoperative clinical course of valvular IE due to *Staphylococcus aureus* is usually significantly worse, suggesting a potential benefit of early surgery in these patients [79,80,81].

Other rarer but emerging causative pathogens of valvular IE that often demonstrate an aggressive clinical course include *Streptococcus agalactiae*, *Pseudomonas aeruginosa*, *Coxiella burnetti*, *Bartonella*, *Brucella* and *Candida albicans*. Left-sided valvular IE due to *Streptococcus agalactiae* is similar in severity to *Staphylococcus aureus*, with comparable rates of HF, paravalvular complications, sepsis, stroke, systemic embolic events and mortality [39]. However, the incidence of acute renal failure is significantly decreased [39]. *Pseudomonas aeruginosa* mostly affects the right-sided heart valves, where medical treatment is usually sufficient [29]. With increasing reports of left-sided valvular IE, a more aggressive clinical course has been reported [40,41]. *Coxiella burnetti*, *Bartonella* and *Brucella*, the frequent causes of culture-negative IE, are associated with complicated courses of valvular IE, including increased rates of HF, valve destruction, abscess formation and persistent infection leading to sepsis [74]. Lastly, valvular IE caused by *Candida albicans* frequently leads to paravalvular complications, bulky vegetations and cerebral and systemic embolic events [74]. Considering early surgery in patients diagnosed with valvular IE caused by any of these pathogens is crucial. 

## 6. Conclusions

Infective endocarditis is a deadly disease that is associated with a significant burden. With a growing elderly population, an increasing incidence of degenerative valvopathies, an increasing use of prosthetic heart valves and an increasing incidence of antibiotic resistances, the incidence of the disease is on the rise. Moreover, the changing epidemiology has also caused the microbiology of IE to evolve. With an increased incidence of IE caused by emerging and re-emerging difficult-to-treat pathogens, growing evidence has shown the potential benefit of early valve surgery instead of medical therapy alone. The establishment of dedicated endocarditis teams has led to improved outcomes. However, more RCTs are needed to continuously progress the management and outcomes of this constantly evolving disease. 

## Figures and Tables

**Table 1:** Current guidelines outlining the recommended empirical antibiotic treatment in adults with suspected infective endocarditis according to the place of infection and the involvement of native or prosthetic heart valves [11].

Clinical Scenario	Empirical Antibiotic Therapy	(Class/Level of Evidence)
Community-acquired NVE or late ^a^ PVE	Ampicillin 12 g/d, i.v. in 4–6 doses	IIa/C
	Ceftriaxone 4 g/d, i.v. or i.m. in 2 doses	
	(Flu)cloxacillin 12 g/d, i.v. in 2 doses	
	Gentamicin 3 mg/kg/d, i.v. or i.m. in 1 dose	
Community-acquired NVE or late ^a^ PVE in patients allergic to beta-lactam antibiotics	Cefazolin 6 g/d, i.v. in 3 doses	IIb/C
	Vancomycin 30 mg/kg/d, i.v. in 2 doses	
	Gentamicin 3 mg/kg/d, i.v. or i.m. in 1 dose	
Nosocomial/Non-nosocomial healthcare associated IE or early ^b^ PVE	Vancomycin 30 mg/kg/d, i.v. in 2 doses	IIb/C
	Daptomycin 10 mg/kg/d, i.v. in 1 dose	
	Gentamicin 3 mg/kg/d, i.v. or i.m. in 1 dose	
	Rifampin 900–1200 mg, i.v. or p.o. in 2 or 3 doses	

^a^ ≥12 months after index procedure; ^b^ ≤12 months after index procedure; IE—infective endocarditis; NVE—native valve endocarditis; PVE—prosthetic valve endocarditis.

**Table 2:** Main indications for surgery in patients diagnosed with infective endocarditis, as outlined in the current American and European guidelines.

Guidelines and Year (Reference)	Recommendations	(Class/Level of Evidence)
ACC/AHA 2020 [10]	Emergency ^a^ surgery recommended in aortic or mitral NVE or PVE with severe acute regurgitation, obstruction or fistula causing refractory pulmonary edema or cardiogenic shock	I/B
	Urgent ^b^ surgery recommended in aortic or mitral NVE or PVE with severe acute regurgitation or obstruction causing symptoms of HF or echocardiographic signs of poor hemodynamic tolerance	I/B
	Urgent ^b^ surgery recommended in locally uncontrolled infection (abscess, false aneurysm, fistula, enlarging vegetation, prosthetic dehiscence, new AVB)	I/B
	Urgent ^b^ or non-urgent surgery recommended in IE caused by fungi or multiresistant organisms according to hemodynamic condition	I/C
	Urgent ^b^ surgery recommended in aortic or mitral NVE or PVE with persistent vegetations ≥ 10 mm after one or more embolic episodes despite appropriate antibiotic therapy	I/B
	Urgent ^b^ surgery recommended in IE with vegetation ≥ 10 mm and other indications for surgery	I/C
ESC 2023 [11]	In patients with IE who present with valve dysfunction resulting in symptoms of HF, early ^c^ surgery is indicated	I/B-NR
	In patients with left-sided IE caused by *S. aureus*, a fungal organism or other highly resistant organisms, early ^c^ surgery is indicated	I/B-NR
	In patients with IE complicated by AVB, annular or aortic abscess, or destructive penetrating lesions, early ^c^ surgery is indicated	I/B-NR
	In patients with IE and evidence of persistent infection, as manifested by persistent bacteremia or fevers lasting >5 days after onset of appropriate antimicrobial therapy, early ^c^ surgery for IE is indicated	I/B-NR
	For patients with PVE and relapsing infection (defined as recurrence of bacteremia after a complete course of appropriate antibiotics and subsequent negative blood culture results) without other identifiable source of infection, surgery is recommended	I/C-LD

^a^ Within 24 h; ^b^ Within 3–5 days; ^c^ During initial hospitalization and before completion of a full therapeutic course of antibiotics; ACC—American College of Cardiology; AHA—American Heart Association; AVB—atrioventricular block; ESC—European Society for Cardiology; HF—heart failure; IE—infective endocarditis; NVE—native valve endocarditis; PVE—prosthetic valve endocarditis.

## Abbreviations

ASDR: age-standardized DALYs rate; ASIR: age-standardized incidence rate; ASMR: age-standardized mortality rate; BCNIE: blood-culture-negative endocarditis; CT: computed tomography; DALYs: disability-adjusted life years; EAPC: estimated annual percentage change; ESC: European Society of Cardiology; HF: heart failure; IE: infective endocarditis; MRI: magnetic resonance imaging; NVE: native valve endocarditis; PCR: polymerase chain reaction; PVE: prosthetic valve endocarditis; SAVR: surgical aortic valve replacement; SDI: socio-demographic index; TAVR: transcatheter aortic valve replacement; TEE: transesophageal echocardiography; TTE: transthoracic echocardiography.
